# Trends in Serum Cytokine Expression in Pediatric Skeletal Dysplasia

**DOI:** 10.1002/jbm4.10816

**Published:** 2023-11-09

**Authors:** David A. O'Connell, Ricki S. Carroll, Angela L. Duker, Andrea J. Schelhaas, Marjorie M. Postell, Paul T. Fawcett, Michael B. Bober

**Affiliations:** ^1^ Thomas Jefferson University Philadelphia PA USA; ^2^ Nemours Children's Hospital, Delaware Wilmington DE USA

**Keywords:** cytokines, diseases and disorders of/related to bone, osteogenesis imperfecta (oi)

## Abstract

The skeletal dysplasias are a heterogeneous group of genetic conditions caused by abnormalities of growth, development, and maintenance of bone and cartilage. Little is known about the roles that cytokines play in the inflammatory and non‐inflammatory pathophysiology of skeletal dysplasia. We sought to test our hypothesis that cytokines would be differentially expressed in children with skeletal dysplasia as compared to typically growing controls. Cytokine levels were analyzed using the Cytokine Human Magnetic 25‐Plex Panel (Invitrogen, Waltham, MA, USA); 136 growing individuals with skeletal dysplasia and compared to a cohort of 275 healthy pediatric control subjects. We focused on the expression of 12 cytokines across nine dysplasia cohorts. The most common skeletal dysplasia diagnoses were: achondroplasia (58), osteogenesis imperfecta (19), type II collagenopathies (11), multiple epiphyseal dysplasia (MED: 9), diastrophic dysplasia (8), metatropic dysplasia (8), and microcephalic osteodysplastic primordial dwarfism type II (MOPDII: 8). Of the 108 specific observations made, 45 (41.7%) demonstrated statistically significant differences of expression between controls and individuals with skeletal dysplasia. Four of the 12 analyzed cytokines demonstrated elevated expression above control levels in all of the dysplasia cohorts (interleukin 12 [IL‐12], IL‐13, interferon γ‐induced protein 10 kDa [IP‐10], regulated on activation, normal T cell expressed and secreted [RANTES]) and two demonstrated expression below control levels across all dysplasia cohorts (monocyte chemoattractant protein 1 [MCP‐1], macrophage inflammatory protein‐1β [MIP‐1β]). The highest levels of overexpression were seen in MOPDII, with expression levels of IP‐10 being increased 3.8‐fold (*p* < 0.0001). The lowest statistically significant levels of expressions were in type II collagenopathies, with expression levels of MCP‐1 being expressed 0.43‐fold lower (*p* < 0.005). With this data, we hope to lay the groundwork for future directions in dysplasia research that will enhance our understanding of these complex signaling pathways. Looking forward, validating these early trends in cytokine expression, and associating the observed variations with trends in the progression of dysplasia may offer new candidates for clinical biomarkers or even new therapeutics. © 2023 The Authors. *JBMR Plus* published by Wiley Periodicals LLC. on behalf of American Society for Bone and Mineral Research.

## Graphical abstract

We identified differences in cytokine expression in multiple types of pediatric skeletal dysplasia as compared to typically growing children. Four of the 12 analyzed cytokines demonstrated elevated expression above control levels in all of the dysplasia cohorts (IL‐12, IL‐13, IP‐10, RANTES) and 2 demonstrated expression below control levels across all dysplasia cohorts (MCP‐1, MIP‐1β).

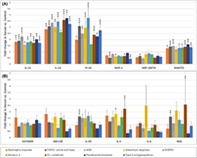

## Introduction

The skeletal dysplasias are a heterogeneous group of genetic conditions caused by abnormalities of growth, development, and maintenance of bone and cartilage. In the most recent version of the Nosology of Genetic Skeletal Disorders published in 2023, there are 771 distinct entries which are classified into 41 groups. Seventy‐two percent of these disorders (552/771) now have a known genetic basis.^(^ ^1^ ^)^ Although cytokines were first functionally characterized in the regulation of inflammation and immune responses, we now know that they are involved in the cellular signaling of nearly every biological process.^(^ ^2^ ^)^ Currently, little is known about the roles that cytokines play in the inflammatory and non‐inflammatory pathophysiology of skeletal dysplasia. Published data regarding the role of various cytokines in the pathogenesis of skeletal dysplasia are sparse, but preliminary data clearly point to activity of cytokines in specific skeletal processes, such as bone remodeling.^(^ ^3^, ^4^ ^)^ In recent years, cytokines have also been implicated as potential prognostic biomarkers in bone disease—specifically, multiple studies have found aberrant levels of inflammatory cytokines in mucopolysaccharidosis‐associated dysplasia.^(^ ^5^, ^6^ ^)^

The potential use of cytokines as biomarkers and targets of therapy in the management of skeletal dysplasia is intriguing. Owing in large part to their wide anatomic distribution and characteristic pleiotropism, it is often difficult to provide comprehensive description of cytokines' effects throughout the body.^(^ ^7^ ^)^ As the literature involving cytokines in disease continues to expand, their value in diagnosis and therapy grows as well. In inflammatory and autoimmune disease, cytokines and cytokine antagonists have already earned indispensable roles in medical management.^(^ ^8^, ^9^ ^)^ Given the availability and clinical efficacy of some of these therapies, there is abundant reason to pursue advancement in the understanding of the complex roles that cytokines play in the pathophysiologies of skeletal dysplasias. The cytokines studied here are known to play roles in inflammation (eg, interleukins 4, 6, 12, and 13) (IL‐4, IL‐6, IL‐12, and IL‐13), granulocyte‐macrophage colony‐stimulating factor (GM‐CSF), and interleukin‐2 receptor (IL‐2R) as well as chemotaxis (eg, interferon gamma induced protein 10 [IP‐10], monocyte chemoattractant protein 1 [MCP1], macrophage inflammatory protein 1β [MIP‐1β], regulated on activation, normal T cell expressed and secreted [RANTES], eotaxin, and monokine induced by gamma interferon [MIG]).^(^ ^10^, ^11^ ^)^

This study was performed as part of an institutional review board (IRB)‐approved study which examined the relationships between skeletal dysplasia and various biomarkers. To date, using the same samples presented in this manuscript, C‐Type natriuretic peptide and Collagen X marker data have been published in the *FGFR3*‐opathies.^(^ ^12^, ^13^ ^)^ To this end, we sought to test our hypothesis that cytokines could be differentially expressed in children with skeletal dysplasia as compared to typically growing controls. Here we present preliminary data from all collected pediatric samples and their serum cytokine expression.

## Subjects and Methods

### Samples

All individuals were seen in the skeletal dysplasia clinic at Nemours Children's Hospital – Delaware and enrolled in a prospective study approved by the Nemours Institutional Review Board for investigation of C‐type natriuretic peptide in skeletal dysplasias. All children had written parental permission obtained. Parental permission forms included authorization to archive and use blood samples for future research studies. Cytokine levels were measured in archived serum samples, collected from April 2011–July 2017, stored at −80°C. Control serum samples were received from Nemours Children's Hospital ‐ Florida Biobank, collected from healthy volunteer children in a study designed to acquire normal pediatric serum samples. Samples were stored in a −80°C freezer until testing.

### Cytokine quantification assay

Cytokine levels were analyzed using the Cytokine Human Magnetic 25‐Plex Panel (Invitrogen, Waltham, MA, USA) according to the manufacturer's instructions and quantified using a Luminex 200 instrument with xPONENT 4.2 software (Luminex, Austin, TX, USA). The test kit allowed for the quantification of IL‐1β, IL‐1RA, IL‐2, IL‐2RA, IL‐4, IL‐5, IL‐6, IL‐7, IL‐8, IL‐10, IL‐12 (p40/p70), IL‐13, IL‐15, IL‐17, tumor necrosis factor α (TNF‐α), interferon α (IFN‐α), IFN‐γ, granulocyte‐macrophage colony‐stimulating factor (GM‐CSF), macrophage inflammatory protein 1α (MIP‐1α), MIP‐1β, IP‐10, MIG, Eotaxin, RANTES, and MCP‐1.

### Statistical analysis

Differential serum cytokine expression among the dysplasia cohorts was presented as a fold change from control expression. Mean and standard error were calculated for each dysplasia cohort. Significance testing was performed via two tailed *t* test between each dysplasia cohort and the nondysplastic control population for each of the qualified cytokines. Three tiers of significance were established to characterize the results of statistical testing (*p* < 0.05; *p* < 0.005; *p* < 0.0001).

## Results and Discussion

### Characteristics of the subjects

Cytokine expression was measured in 136 growing individuals with skeletal dysplasia (mean age 7.5 years, median age 6.9 years, range 0.3–17.6 years, 79 males/57 females) and compared to expression in a cohort of 275 nondysplastic control patients (mean age 12.4 years, median age 12.7, range 1.9–19.8 years, 150 males/125 females). The most common diagnoses were: achondroplasia (58), osteogenesis imperfecta (OI: mild, 3; moderate, 9; severe, 7), type II collagenopathies (11), multiple epiphyseal dysplasia (MED: 9), diastrophic dysplasia (8), metatropic dysplasia (8), microcephalic osteodysplastic primordial dwarfism type II (MOPDII: 8), hypochondroplasia (6), Morquio syndrome type A (5), and pseudoachondroplasia (4). Age and gender information by group is shown in Table 1.

**Table 1:** Demographics

Characteristic	Number in cohort (*n*)	Mean age (years)	Median age (years)	Age range (years)	Male:Female (*n*:*n*)
All dysplasias	136	7.5	6.9	0.3–17.6	79:57
Controls	275	12.4	12.7	1.9–19.8	150:125
Achondroplasia	58	6.1	4.6	0.3–17.3	31:27
OI	19	9.1	9.0	2.0–17.0	11:8
Mild	3	8.4	9.9	3.0–12.3	2:1
Moderate	9	9.6	9.0	4.2–14.1	5:4
Severe	7	8.7	8.2	2.0–17.0	4:3
Type II collagenopathy	11	9.4	10.1	1.4–16.0	5:6
MED	9	7.1	5.2	4.3–12.7	8:1
Diastrophic dysplasia	8	6.5	6.8	1.0–13.7	5:3
Metatropic dysplasia	8	10.7	12.5	1.5–17.6	4:4
MOPD II	8	6.9	7.7	0.3–11.2	4:4
Hypochondroplasia	6	8.7	8.6	6.3–11.4	3:3
Morquio A	5	7.2	5.7	0.9–17.4	4:1
Pseudoachondroplasia	4	12.0	12.0	8.0–15.9	4:0

### Cytokine inclusion

Data acquired from 12 of the 25 measured cytokines was returned in sufficiently high volume and quality to warrant analysis—inclusion criteria for statistical analysis were a minimum 40% quantitative assay yield among all individual dysplasia cohorts and among control samples. The total quantitative yields for included cytokines among control samples and dysplasia cohorts were 92% and 95%, respectively.

### Cytokine expression

**Fig. 1:**
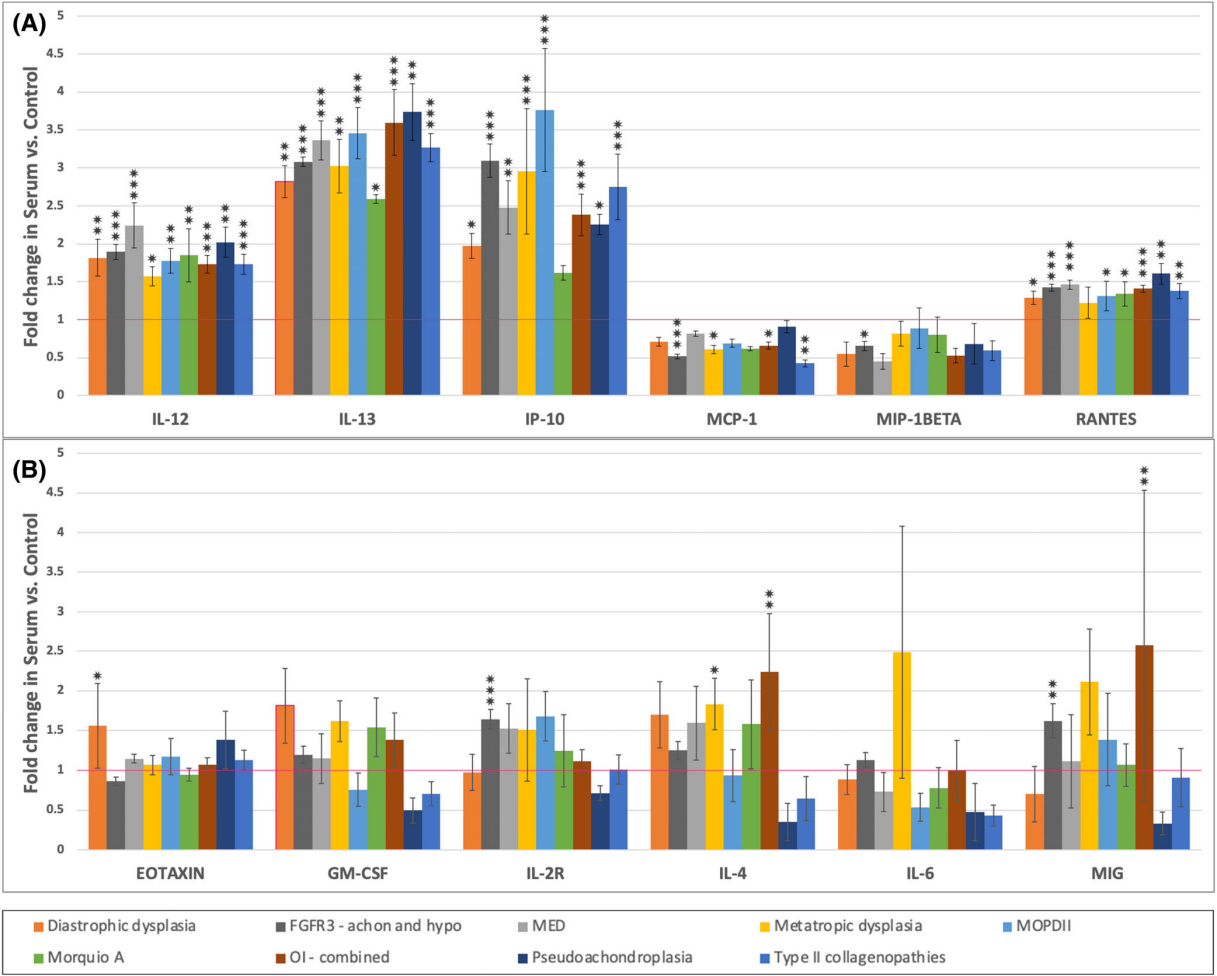
(*A*) Cytokines with consistently elevated or consistently decreased expression across all the skeletal dysplasia cohorts. (*B*) Cytokines with variable expression across skeletal dysplasia cohorts. Expression is measured as a fold change from the control population (control expression represented by horizontal red line). Standard error is represented by error bars. Significance testing between dysplasia cohorts and control via two tailed *t* test is represented by *p < 0.05, **p < 0.005, and ***p < 0.0001.

Four of the 12 analyzed cytokines demonstrated elevated expression above control levels in all of the dysplasia cohorts (IL‐12, IL‐13, IP‐10, RANTES) and two demonstrated expression below control levels across all dysplasia cohorts (MCP‐1, MIP‐1β) (Fig. 1*A*). Six cytokines (Eotaxin, GM‐CSF, IL‐2R, IL‐4, IL‐6, MIG) yielded variable expression differences across the dysplasia groups when compared to controls (Fig. 1*B*). The highest levels of overexpression were seen in MOPDII, with expression levels of IP‐10 being increased 3.8‐fold (*p* < 0.0001). The lowest statistically significant levels of expressions were in type II collagenopathies, with expression levels of MCP‐1 being expressed 0.43‐fold lower (*p* < 0.005).

There were 40 combinations of cytokines and dysplasia cohorts which reached statistically significant increased expression patterns. All nine cohorts demonstrated IL‐13 and IL‐12 overexpression. Eight of nine dysplasia cohorts demonstrated increased RANTES and IP‐10 expression. Specific increased expression differences were noted in IL‐2R and MIG within the *FGFR3* cohort (achondroplasia and hypochondroplasia), in IL‐4 and MIG within the OI cohort, in Eotaxin within the diastrophic dysplasia cohort, and in IL‐4 within the metatropic dysplasia cohort.

Only five combinations of cytokines and dysplasia cohorts yielded results involving statistically significant decreased serum expression. OI, metatropic dysplasia, *FGFR3‐*opathies, and type II collagenopathies all showed decreased expression of MCP‐1. *FGFR3*‐opathies further showed statistically significant decreased expression of MIP‐1β.

### Interpretation

The understanding of the role played by various cytokines in the physis, articular cartilage, and bone in pediatric growth and development is poorly understood. This first of its kind exploratory study, however, allows for the identification of specific molecules on which future hypotheses and experiments can be based. We focused on the expression of 12 cytokines across nine dysplasia cohorts. Of the 108 specific observations made, 45 (41.7%) demonstrated statistically significant differences of expression between controls and individuals with skeletal dysplasia. At this time, the multiple overlapping effects of any specific cytokine obscures clear functional categorization in the setting of skeletal dysplasia. IL‐13 (upregulated in nine cohorts) and IL‐4 (upregulated in the OI and metatropic dysplasia cohorts) are well categorized for their complex roles promoting type II inflammation, but for many years they have also been known to inhibit bone resorption through suppression of prostaglandin synthesis.^(^ ^14^ ^)^ IL‐12 (upregulated in nine cohorts) is known to indirectly inhibit osteoblastogenesis.^(^ ^15^ ^)^ RANTES (upregulated in eight cohorts) has been identified as a likely factor in the development of osteoarthritis.^(^ ^16^ ^)^ IP‐10 (upregulated in eight cohorts) has been negatively correlated with bone mineral density in patients with dermatomyositis.^(^ ^17^ ^)^ The majority of identified cytokines are recognized to have some relationship to the musculoskeletal system, albeit in signaling pathways which are early in their elucidation stage.

### Limitations

Notably, one limitation of the dataset was the sample size of the dysplasia cohorts. The largest dysplasia cohort by a significant margin was the *FGFR3* cohort, which included 58 individuals with achondroplasia and six with hypochondroplasia; the power afforded by a larger sample size was notable in the high frequency of statistically significant *p* values observed in this cohort. Another limitation is that there are no well‐established normal ranges for cytokine expression in typically growing and developing children. A larger scale study comprising all of the pediatric skeletal dysplasia cohorts with expanded sample sizes and improved controls more precisely matched to age and sex could provide reinforcement of these preliminary results.

## Conclusions

In summary, we were able to confirm our hypothesis that cytokines are differentially expressed in children with skeletal dysplasia as compared to typically growing children. We focused on the expression of 12 cytokines across nine dysplasia cohorts. Of the 108 specific observations made, 45 (41.7%) demonstrated statistically significant differences of expression between controls and individuals with skeletal dysplasia. IL‐12, IL‐13, IP‐10, and RANTES expression was higher in all dysplasia cohorts as compared to controls. MCP‐1 and MIP‐1β were decreased in all dysplasia cohorts as compared to controls. With this data, we hope to lay the groundwork for future directions in dysplasia research that will enhance our understanding of these complex signaling pathways. Looking forward, validating these early trends in cytokine expression, and associating the observed variations with trends in the progression of dysplasia may offer new candidates for clinical biomarkers or even new therapeutics.

## Author Contributions

**David O'Connell:** Data curation; formal analysis; writing – original draft; writing – review and editing. **Ricki Carroll:** Data curation; investigation; supervision; writing – original draft; writing – review and editing. **Angela L. Duker:** Supervision; writing – original draft; writing – review and editing. **Andrea Schelhaas:** Supervision; writing – original draft; writing – review and editing. **Marjorie Postell:** Data curation; formal analysis; investigation; methodology; software; writing – review and editing. **Paul Fawcett:** Conceptualization; formal analysis; investigation; methodology; resources; software; supervision; validation; writing – review and editing. **Michael B. Bober:** Conceptualization; funding acquisition; investigation; project administration; supervision; writing – original draft; writing – review and editing.

## Disclosures

The authors declare they have no conflicts of interest to disclose.

### Peer Review

The peer review history for this article is available at https://www.webofscience.com/api/gateway/wos/peer‐review/10.1002/jbm4.10816.

## Data Availability

Data will be made available upon request.
